# A simple diabetes vascular severity staging instrument and its application to a Torres Strait Islander and Aboriginal adult cohort of north Australia

**DOI:** 10.1186/1472-6963-12-185

**Published:** 2012-07-03

**Authors:** Odette R Gibson, Leonie Segal, Robyn A McDermott

**Affiliations:** 1 Health Economics and Social Policy Group, Division of Health Sciences, University of South Australia, Adelaide, 5001, Australia; 2 Sansom Institute, Division of Health Sciences, University of South Australia, Adelaide, 5001, Australia

**Keywords:** Diabetes vascular staging instrument, Aboriginal and Torres Strait Islander Australians

## Abstract

**Background:**

To develop an instrument that predicts diabetes-related vascular disease severity using routinely collected data on Australian Aboriginal and Torres Strait Islander adults with type 2 diabetes, in the absence of diabetes duration.

**Methods:**

A complex diabetes severity classification system was simplified and adapted for use with an Australian Aboriginal and Torres Strait Islander adult population with type 2 diabetes in north Queensland. Detailed vascular health risks and morbidities were mapped to routinely collected measures. Individual–level health screening, hospital separation and mortality data were linked and used to plot mean monthly in-patient hospital cost and percent mortality by disease severity as defined by the newly developed instrument, to test construct validity.

**Results:**

The revised instrument consists of four combined diabetes-related microvascular and macrovascular stages that range from least severe (stage 1) to severe irreversible vascular impairment (stage 4). When applied to data of an Aboriginal and Torres Strait Islander Australian population the instrument showed good construct validity, predicting higher hospital cost and mortality as vascular disease severity increased.

**Conclusions:**

This instrument discriminates between levels of diabetes-related vascular disease severity, displays good construct validity by predicting increased hospital cost and mortality with worsening severity and can be populated with routinely collected data. It may assist with future health service research and its use could be extended to practice settings for health care planning for diabetes management programs and monitoring vascular disease progression.

## Background

Type 2 diabetes is one of the most common non-communicable diseases in the world and results in substantial morbidity and premature mortality [1]. The incidence and prevalence of diabetes is rising in developed and developing countries, the age of onset is becoming younger in adults and prevalence of type 2 diabetes in children is increasing [2]. Diabetes is a contributing factor to cardiovascular disease [3] and a leading cause of end stage renal failure [4]. These complications are a great burden to patients and society [5], incur high health service use and contribute excessively to the cost of health care [6].

In evaluating the impact of primary health care resourcing on hospital use and mortality in a high risk population of Australian Indigenous adults with type 2 diabetes it was necessary to control for the potential confounding of diabetes-related health status. Given a relatively small sample cohort (n = 379) and no reliable estimate of diabetes duration, a simple measure of diabetes-related health status was sought. We adapted a complex vascular classification system developed for an Australian diabetes coordinated care trial to our study population.

A Trial of Integrated Care for persons with Diabetes commenced in 1996 in three pilot sites across Australia, including the mid-north coast New South Wales (NSW). An objective of the trial was to document the costs of management over two years. Annual health care cost per person, by disease severity was estimated [7]. An expert clinical group informed the development of a vascular staging system to classify diabetes according to clinical severity. The resulting severity classification system consisted of four microvascular and four macrovascular disease stages based on 44 detailed comorbidities [7]. The four microvascular stages followed diabetes-related vascular disease progression from mild to severe irreversible microvascular impairment and likewise the four macrovascular stages. It had a dual classification staging system that assigned each individual to one of 16 possible joint micro/macrovascular stages (micro 1–4; macro 1–4). The most severe microvascular and macrovascular stage met determined an individuals’ vascular score. Available data on trial participants sourced from the trial developed clinical/audit management form, hospital in-patient and outpatient services, general practice services and pharmaceutical records held by the Health Insurance Commission were used to populate the instrument. Results of the economic analysis showed an increase in mean annual cost of inpatient services at every disease stage, across both microvascular and macrovascular stages, for the NSW population with diabetes.

Our objective was to revise the NSW diabetes classification system to arrive at a simple diabetes vascular severity staging instrument that used only data collected routinely in health practice settings, plus hospitalisation data. This paper describes the revisions made to the NSW instrument, the method used to match health screening data with hospital separation and death data, and the performance of the new instrument mapped against hospital cost and mortality when applied to our study population.

## Methods

### Defining microvascular and macrovascular disease stages

A microvascular complication was defined as early signs of diabetic nephropathy or evidence of established diabetic nephropathy or retinopathy. Macrovascular complications captured clinical risks of acquiring, or evidence of, cardiovascular disease, peripheral vascular disease or cerebrovascular vascular disease. Generally an early sign of a vascular complication was determined by results of a health check (abnormal lipid profile, high blood pressure), and evidence of a vascular complication was determined by being hospitalised for the condition.

### Revisions to the existing vascular staging instrument

Microvascular and macrovascular detailed classification items were combined within their broad vascular stages. Vascular stage 1 is defined by absence of microvascular disease or macrovascular risk factors (but allows for the presence of lifestyle risk factors, like obesity and smoking, which while risk factors for diabetes are not *per se* evidence of disease progression). Screen-detected microvascular comorbidities and risk factors for macrovascular disease were combined and form vascular stage 2. Likewise moderate microvascular and macrovascular comorbidities were combined to create one vascular stage (stage 3) and late-stage micro and macro vascular comorbidities were combined to form vascular stage 4.

Albumin/creatinine ratio (ACR) was chosen as a measure of the early sign of diabetic nephropathy. Proteinuria in the original instrument was replaced by macroalbuminuria and microalbuminuria (measured by urinary ACR) for the ability to differentiate between levels of kidney function [8]. Elevated cholesterol >5.5 mmol/L was replaced by elevated triglycerides >1.5 mmol/L due to evidence that dyslipidemia in the Australian Aboriginal and Torres Strait Islander population is characterised by a lipid profile of elevated triglycerides levels >1.5 mmol/L and reduced high density lipoprotein (HDL) cholesterol <1.0 mmol/L rather than elevated total cholesterol [9].

Detailed classification items not routinely collected for annual health checks or hospital separation were excluded. Items excluded were stroke without residual deficit, background retinopathy on retinal examination, abnormal peripheral sensation, areas of dried skin on the feet or distinct foot callus present on examination, ankle reflexes absent and abnormal distal foot impulse and serum creatinine. Self-reported smoking was excluded from the staging because it is not evidence of vascular disease*.* Smoking would be included as a distinct variable in our statistical models. Table 1 details inclusion criteria for the new instrument with 4 stages.

**Table 1:** Revised type 2 diabetes vascular staging instrument with detailed classification items and their measures

*Vascular stage & broad classification of stages*		
	Detailed classification item	Measures (Australian Refined Diagnosis Related Group code version 5.0^*^ or clinical reference range)
*1*	*Diagnosis of type 2 diabetes*	Doctor diagnosis confirmed by a medical chart audit or a fasting blood glucose level ≥ 7 mmol/L^†^ or 2-hour glucose tolerance test result of blood glucose level ≥ 11.1 mmol/L ^†^[10] or a hospital admission for diabetes
	*With no evidence of microvascular or macrovascular risk factors (but may be obese or report current tobacco smoking)*	
	Albumin creatinine ratio (ACR) within normal range	ACR of 0–2.5 mg/mmol for men and 0–3.5 mg/mmol for women^‡^
	Normal high density lipoprotein cholesterol (HDLC)	> 1.0 mmol/L^§^
	Normal triglycerides	< 1.5 mmol/L^§^
	Not hypertensive	Not hospitalised for hypertension (DRG code F67A or F67B), or blood pressure < 140/90 mmHg ^||^
	No evidence of retinopathy	Not hospitalised for retinal procedures and vascular disorders of the eye (DRG code C03Z or C16Z)
	Peripheral sensation normal and no evidence of claudication	Not hospitalised for DRG code F65A or F65B
	No cardiac disease	Not hospitalised for DRG code A05Z, F12Z, F14A, F14B, F62Z, F62B, F70A, F70B, F62A, F14C, F15Z, F16Z, F17Z, F18Z, F19Z, F10Z, F41A, F41B, F60A, F60B or F60C
*2*	*Screen-detected microvascular comorbidities and/or risk factors for macrovascular disease*	
	Microalbuminuria	ACR 2.6–25 mg/mmol for men and 3.6-35 mg/mmol for women^‡^
	Reduced HDLC	≤ 1.0 mmol/L^§^
	Elevated triglycerides	≥ 1.5 mmol/L^§^
	Hypertension	Hospitalised for DRG code F67A or F67B, or systolic blood pressure ≥ 140 mm Hg and/or a diastolic blood pressure ≥ 90 mm Hg^||^
*3*	*Moderate microvascular and/or macrovascular comorbidities*	
	Macroalbuminuria	ACR > 25 mmol/L for men and > 35 mmol/L for women^‡^
	History of coronary artery bypass graft or angioplasty	F05A, F05B, F06A, F06B
	History of carotid artery disease	F66A, F66B
	History of or hospitalised for angina	F72A, F72B
	History of claudication or peripheral vascular disease	F65A, F65B
*4*	*Late-stage diabetes macrovascular and/or microvascular comorbidities*	
	Amputation of lower limb	F11A, F11B, F13Z, I07Z, I14Z
	Present or history of gangrene of the lower limb	J13A, J13B, J64A, J64B
	History of stroke with residual deficit	B69A, B69B, B70A, B70B, B70C, B70D
	History of or hospitalised for cardiac failure	A05Z, F12Z, F14A, F14B, F62A, F62B, F70A, F70B
	History of myocardial infarction	F14C, F15Z, F16Z, F17Z, F18Z, F19Z, F10Z, F41A, F41B, F60A, F60B, F60C
	Proliferative retinopathy and both eyes: only able to perceive light or hand movement or count fingers, or unable to perceive light	C03Z, C16Z
	Present or history of osteomyelitis or ulcer of the foot or charcot joint	I64A, I64B
	Bacterial soft tissue infection of the foot	J12A, J12B, J12C,
	History of painful neuropathy or autonomic neuropathy other than erectile impotence	K01Z
	History of end-stage renal failure	L60A, L60B, L60C
	History of dialysis	L61Z
	History of renal transplant	A09A, A09B

Sources:

^*^ Department of Health and Aging: Public Sector - Estimated Round 9 (2004–05) AR_DRG 5.0 Cost Report.

[http://www.health.gov.au/internet/main/publishing.nsf/Content/88F4E78E15620A80CA2571CB0004DDAA/$File/_R9CWQLDEst.pdf]

^‡^Diabetes Australia’s Health Care and Education Committee and RACGP's National Standing Committee for Quality Care in 2010: Diabetes management in general practice. [http://www.racgp.org.au/Content/NavigationMenu/ClinicalResources/RACGPGuidelines/Diabetesmanagement/200910diabetesmanagementingeneralpractice.pdf]

^||^ National Blood Pressure Advisory Committee: Hypertension management guide for doctors 2004. [http://www.sld.cu/galerias/pdf/servicios/hta/hypertension_management_guide_australia_2004.pdf]

^§^National Heart Foundation of Australia and Cardiac Society of Australia and New Zealand: Lipid management guidelines-summary paper. *Medical J Aust 2*001, **175:** S57-S88.

^†^World Health Organization: Part 1: diagnosis and classification of diabetes mellitus, in: definition, diagnosis and classification of diabetes mellitus and its complications. [http://www.staff.ncl.ac.uk/philip.home/who_dmg.pdf]

### Mapping objective measures to detailed classification items

Current reference ranges for screen-detected measures were mapped to relevant detailed classification items and referenced. Australian Refined Diagnosis Related Group Version 5.0 (AR-DRGv5.0) codes were mapped to the remaining detailed classification items by one of the authors (RM).

### Data sources

A cross sectional population health screen known as the Well Persons Health Check (WPHC) was conducted in 26 communities in north Queensland Australia. Ethics approval was granted by Cairns Base hospital, Queensland Health. A detailed WPHC study protocol is published elsewhere [11]. Briefly, screenings commenced in March 1998 for a period of one week in each community and were completed in December 2000. People aged 13 years and over volunteered to participate (n = 3507). Demography, anthropometric and biochemical measures and self-reported lifestyle behaviors were collected with the primary purpose of early detection and intervention in relation to chronic diseases. This study cohort is adults aged 15 years and over who participated in the WPHC study with a medical diagnosis of type 2 diabetes (n = 379) prior to or at the time of their health check (between Mar 1998 and Dec 2000).

Hospital separation data on consenting WPHC participants from eight public hospitals for the period 25 May 1997 to 31 December 2005 were collected. These are the major referral hospitals for the communities. Death data were sought from the Queensland State Registrar Office of Births, Deaths and Marriages [12].

### Data linkage

Deterministic record linkage based on hospital unit record numbers was used to match individual health screen to hospital separation data and death data were matched manually. A match protocol was developed. A random sample technique was used to validate the match protocol, comparing the clinical and demographic features of the health screening client record with those of the hospital record. Information was also sourced from Royal Flying Doctor Service evacuation notes, clinical diagnoses, or other notes to reach surety that the clients in the health screen and the hospital record were the same person. A potential match was considered false when the potential match could not be confirmed within the patient hospital record.

Eight years of hospital separation data spanning across 3 diagnosis related group (DRG) versions (3.1 (1995–1997) and 4.0 (1998–2001)) were mapped to A-R DRGv5.0 (2002–2006) [13] with assistance from the Classification Management and Statistics Section Healthcare Services Information Branch, Acute Care Division of the Australian Department of Health and Aging using the DrGroup, Australian Refinement DRG Grouper, PC Version 6.0 (Batch), Visasys Pty Ltd Canberra.

### Instrument application

The WPHC data were analysed to find patients with a diagnosis of diabetes and each case identified was assigned to one of the four vascular stages using the criteria defined in Table 1. Assignment to vascular stage 1 requires the absence of microvascular complications and macrovascular risk factors identified in stages 2 to 4. Assignment to vascular stages 2, 3 or 4 occurs when one or more detailed classification items within a stage is present. Individuals are assigned to the highest vascular stage they meet.

### Analysis

The vascular disease severity score was mapped against mean in-patient hospital cost and death to test how the staging allocation performed. Hospital costs were accrued for each individual from the date of assignment of the vascular score (vascular date) to date of death or 31 December 2005. Hospital cost was defined by bed day adjusted DRG cost for each hospital admission (patient length of stay/mean DRG length of stay * DRG cost weight * 2004/05 DRG dollar value - $2989) using 2004/05 Queensland estimated hospital cost report [14]. To arrive at the mean monthly hospital cost the sum of bed day adjusted DRG costs for each individual was divided by the number of months between their vascular date and 31 December 2005 or date of death. Total in-patient hospital cost of each individual within each stage was summed and divided by the number of people in the vascular stage. Mean monthly in-patient hospital costs were presented by vascular stage and confidence intervals were derived by bootstrapping. At each vascular stage percent of persons who died was calculated. Construct validity was assessed by testing for the expected relationship between the new diabetes severity score and hospitalisation and mortality. All analyses were performed using Stata for Windows Version 10 (StataCorp, 1984–2007).

## Results

Revisions to a complex vascular severity dual classification system have resulted in a simplified instrument with four stages. The instrument comprises vascular risks and conditions that are derivable from routinely collected primary health care and hospital data (Table 1). When applied the instrument reports a single individual score of vascular disease severity. Table 2 shows the study cohort characteristics. In general, compared to non-Indigenous Australians with diabetes, there are significant differences. The study cohort is younger, more females than males have diabetes, and a higher percentage has macroalbuminuria. Mean BMI is similar, a higher percentage of the study cohort have well controlled blood pressure (<140/90 mmHg) but not as well controlled as a national cohort of Indigenous adults with diabetes [15]. During the 5 year follow-up period 60 % of the study cohort was hospitalised and 12 % percent became deceased. Diabetes duration was not documented for two thirds of this study cohort and unreliable for the remaining one third.

**Table 2:** Characteristics of the study cohort (N = 379)

Mean age (years) (SD) (range)	48 (12.5) (17 – 85)
Proportion female	58 %
Mean body mass index (kg/m2)^*^ (n = 376) (SD) (range)	32 (6.4) (16.2 – 52.4)
Waist circumference men <102 cm^*^	33 %
Waist circumference women <88 cm^*^	4 %
Fasting blood glucose level < 5.5 mmol/L^†^ (n = 373)	3.5 %
Blood pressure <140/90 mmHg^‡^ (n = 378)	51.8 %
Macroalbuminuria^†^ (n = 356)	34.5 %

Source: Well Persons Health Check, north Queensland, 1998 – 2000.

^*^National Health and Medical Research Council: Clinical practice guidelines for the management of overweight and obesity in adults. [http://www.health.gov.au/internet/main/publishing.nsf/Content/

http://7AF116AFD4E2EE3DCA256F190003B91D/$File/adults_contents.pdf]

^†^Diabetes Australia's Health Care and Education Committee, RACGP's National Standing Committee for Quality Care in 2010: Diabetes management in general practice. [http://www.racgp.org.au]

^‡^National Blood Pressure Advisory Committee: Hypertension management guide for doctors 2004. [http://www.sld.cu/galerias/pdf/servicios/hta/hypertension_management_guide_australia_2004.pdf]

**Figure 1:**
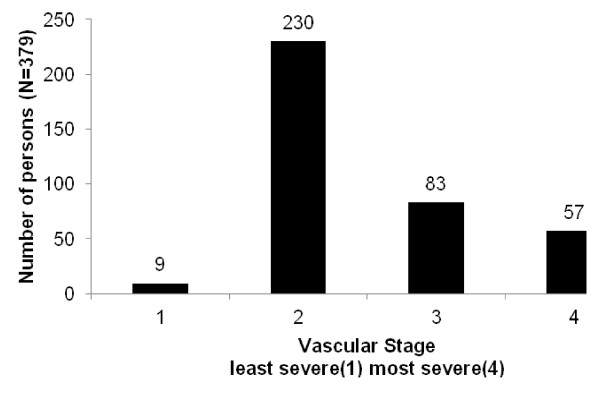
**The distribution of the study population by vascular stage**. Number of people by vascular stage after applying the simplified vascular staging instrument using data from a cohort of Aboriginal and Torres Strait Islander adults with type 2 diabetes who participated in the Queensland Well Persons Health Check (25 May 1997–31 December 2000). N = 379. Vascular stage one (n = 9) is no evidence of microvascular comorbidity and macrovascular risk factors, stage two (n = 230) is presence of screen-detected microvascular comorbidities or risk factors for macrovascular disease, stage three (n = 83) is presence of moderate microvascular or macrovascular comorbidities, stage four (n = 57) is the presence of late-stage macrovascular or microvascular comorbidities.

**Figure 2:**
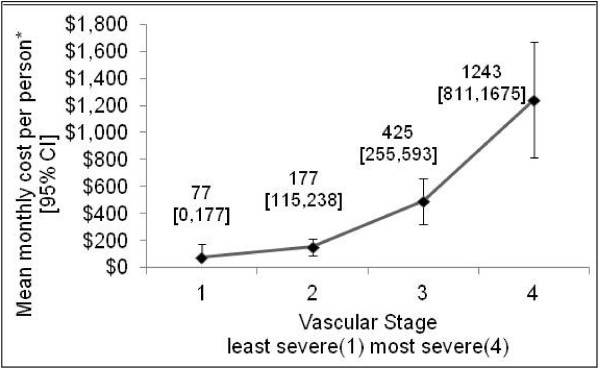
**Mean monthly in-patient hospital cost per person by vascular stage.** Mean monthly in-patient hospital cost (04/05 Australian dollars*) by vascular stage after applying the simplified vascular staging instrument using the public sector Queensland estimated round 9 cost report 2004–05 and hospital admission data from eight Queensland public hospitals (25 May 1997–31 Dec 2005) on a cohort of Aboriginal and Torres Strait Islander adults with type 2 diabetes who participated in the Queensland Well Persons Health Check (1998–2000). N = 379. Hospital cost is derived from the date of assignment to their vascular stage to date of death or 31 December 2005.

**Figure 3:**
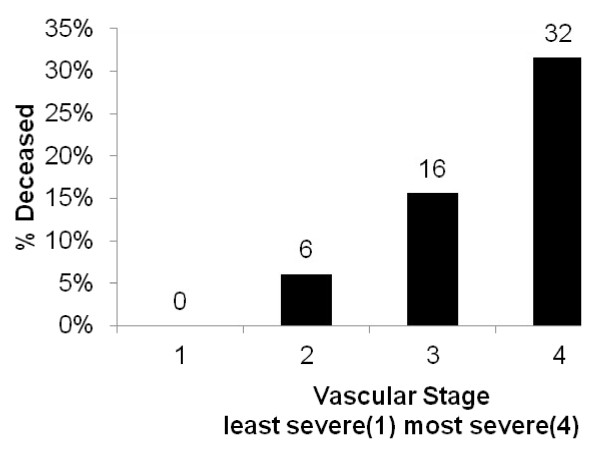
**Percent of persons deceased by vascular stage**. Percent of people deceased by vascular stage after applying the simplified vascular staging instrument using data from a cohort of adults with type 2 diabetes who participated in the Queensland Well Persons Health Check (25 May 1997–31 December 2000) and registered deaths data from Queensland government births, deaths and marriages registry (2000–05). N = 379. No deaths were registered during the period 2000–2005 for participants assigned to vascular stage 1. In total 12 % of the study cohort became deceased subsequent to their health check to 31 December 2005.

Figure 1 shows the distribution of the study population by vascular stage. Only 2 % of the study cohort was in stage 1 (no evidence of microvascular comorbidity and macrovascular risk factors). Most participants (61 %) presented in stage 2 (screen detected microvascular complications or with risk factors for macrovascular disease), 22 % of participants were in stage 3 and 15 % in stage 4 (end-stage micro and macro vascular complications). As found in other studies, in-patient hospital cost [16] and mortality [17] increased with each increase in vascular disease severity, meeting out test for construct validity. Figure 2 shows the mean monthly in-patient hospital cost for each stage, reported in Australian 2004–05 dollars. The mean monthly in-patient hospital cost (±SEM) for stage 2 was, $177 ± $31, over twice that of stage 1, $77 ± $51; stage 3 ($425 ± $86) was five and a half times that of stage 1 and for persons in stage 4 ($1243 ± $220) it was sixteen times that of persons in stage 1. Figure 3 shows the percent of people who died during the 5 year follow-up period by vascular stage. The case fatality rate for this cohort is 12 % for the 5 year period, with the death rate increasing monotonically by vascular stage, (from 0 % in stage 1, 6 % in stage 2, 16 % in stage 3 and 32 % in stage 4).

## Discussion

The simplified vascular staging instrument produced a single diabetes severity score which has shown to predict hospitalisation and mortality and can be applied using only data collected routinely in health practice settings including hospital admissions. It has good construct validity shown by a positive association between disease severity and hospital cost and disease severity and mortality (Figures 2, 3). The distribution of disease severity reflects a common observation in clinical services that the majority of people when diagnosed with diabetes are already in stage 2, having already accumulated a degree of vascular damage (Figure 1).

It is unknown if this instrument would have good construct validity when applied to data of other population groups. The sample size is small, the cohort is young and Indigenous Australians have a lipid profile which differs from that of non-Indigenous Australians [9]. However given the structure of the instrument, there is no reason to presume it will not prove useful in other populations.

A strength of the instrument is its use of routinely collected data from robust sources. Complex testing is not necessary to estimate vascular stage, which can be costly and physically invasive, repetitive and time consuming for participants. Annual and condition specific health checks are becoming core business of primary health care services and hospital separation data is routinely collected in many countries, which can be linked to other population data. The feasibility of this type of research will be enhanced with the adoption of electronic data linkage, which is becoming more common, following its success in Sweden [18], Western Australia [19] and Canada [20]. With future increased availability of linked primary health care and hospital datasets, this instrument could be used more widely and at low cost.

A benefit of a staged disease severity approach is its ability to assign disease severity according to clinical vascular changes, rather than relying on date of diagnosis. Compelling evidence suggests that vascular changes begin to occur years prior to diabetes diagnosis [21]. Harris et al., 1992 [21] studied two groups of patients with diabetes, in the United States and Australia, and found that on-set of diabetic retinopathy was likely to have occurred 4–7 years before clinical diagnosis of diabetes. Furthermore other data in their study suggested that on-set of diabetes may in fact occur 9–12 years before clinical diagnosis. The staged disease severity approach based on clinical markers could capture vascular changes that may occur prior to diagnosis and potentially adjusts for the effect of lifestyle changes and medical and pharmacological advances in diabetes management that may prevent or slow diabetes-related vascular disease progression. In this respect a vascular staging instrument may be more sensitive than diabetes duration as a proxy for health status.

Comparing the performance of the NSW instrument and the simplified version was not feasible, given the more extensive data requirements of the more complex NSW instrument. Two existing severity indexes that use administrative data were found, one did not include the use of screen-detected micro/macrovascular risks[22], and both when applied derived severity scores within complication categories [22,23]; this was considerably more complex than we required. Professionals in collaboration [22] developed a diabetes complication severity index (DCSI) consisting of a 13 point scale with 7 complication categories. Rosenzweig and colleagues [23] index produces 6 separate scores each with 4 levels of severity. Both indexes discussed [22,23] were developed to assist health organizations identify care intensity to provide targeted interventions based on diabetes disease stage, which requires a more finely tuned staging instrument than our purpose of adjusting for heath status as a possible confounder.

Clinical trials have shown that well managed type 2 diabetes can slow the rate of progression of diabetes-related vascular complications reducing the incidence of vascular events [24,25]. However this promise is not always realised in primary care practice settings. One aim of primary health care services is the early detection of diabetes (and other chronic diseases) and early implementation of management to keep people in vascular severity stages one (no evidence of microvascular or macrovascular risk) or two (screen detected microvascular comorbidities or macrovascular risk factors) and prevent progression to more severe stages (irreversible vascular impairment). A clear association between the degree of vascular severity, hospital cost [26] and mortality [17] is established in the literature. Therefore the returns of slowing or preventing diabetes related vascular disease progression should reduce diabetes-related hospital costs and mortality, and enhance the quality of life of patients and their families.

The practical application of this instrument could be within population health research groups and health care delivery settings. At a population level the instrument may be used to inform health services planning for instance by identifying the distribution of vascular severity within a population group at a given point in time. Knowing the percent of people in each disease severity stage may help inform targeted planning of service requirements. In addition, applying the instrument repeatedly over time could allow the rate of diabetes-related vascular disease progression to be calculated and compared over time and across groups. With the inclusion of hospital separations the instrument could also be used to monitor how well a primary care program is performing in managing diabetes.

We propose to use the diabetes vascular severity staging instrument to derive a single severity score which will be used in statistical analysis as a proxy for health status of Indigenous Australian adults with type 2 diabetes. Validating the instrument in future research and testing its generalisability with similar and diverse population groups would be desirable.

## Conclusions

Many population groups are experiencing an increase in type 2 diabetes incidence and prevalence. This instrument discriminates between levels of diabetes-related vascular disease severity, displays good construct validity by predicting increased hospital cost and mortality with worsening severity and can be populated with routinely collected data. It may assist with future health service research, particularly in its current form, when working with small Australian Aboriginal and Torres Strait Islander cohorts. Its use could be extended to practice settings for health care planning for diabetes management programs and monitoring vascular disease progression.

## Competing interests

The authors declare that they have no competing interests.

## Authors’ contributions

O.G. revised the existing instrument, researched data, contributed to the discussion, analyzed the data and drafted the manuscript. L.S revised the existing instrument, designed the study, contributed to the discussion and reviewed/edited the manuscript. R.M. revised the existing instrument, researched data, contributed to the discussion and critically reviewed the manuscript for important intellectual content. All authors read and approved the final manuscript.

## Pre-publication history

The pre-publication history for this paper can be accessed here:

http://www.biomedcentral.com/1472-6963/12/185/prepub
